# Development of Stimuli-Responsive Chitosan/ZnO NPs Transdermal Systems for Controlled Cannabidiol Delivery

**DOI:** 10.3390/polym13020211

**Published:** 2021-01-08

**Authors:** Julia Radwan-Pragłowska, Łukasz Janus, Marek Piątkowski, Aleksandra Sierakowska, Ernest Szajna, Dalibor Matýsek, Dariusz Bogdał

**Affiliations:** 1 Department of Biotechnology and Physical Chemistry, Faculty of Chemical Engineering and Technology, Cracow University of Technology, Warszawska 24 Street, 31-155 Cracow, Poland; j.radwan@doktorant.pk.edu.pl (J.R.-P.); lukasz.janus@doktorant.pk.edu.pl (Ł.J.); a.sierakowska3530@doktorant.pk.edu.pl (A.S.); pcbogdal@cyf-kr.edu.pl (D.B.); 2 Nano Prime sp. z o. o., Metalowców 25, 39-200 Dębica, Poland; e.szajna@uce.com.pl; 3 Faculty of Mining and Geology, Technical University of Ostrava, 70800 Ostrava, Czech Republic; dalibor.matysek@vsb.cz

**Keywords:** chitosan, drug delivery, cannabidiol

## Abstract

One of the most common neurological diseases is epilepsy, which not only negatively affects the quality of people’s life but also may lead to life-threatening situations when its symptoms such as seizures cannot be controlled medically. A very serious problem to be overcame is the untreatable form of this disease, which cannot be cured by any currently available medicines. Cannabidiol, which is a natural product obtained from *Cannabis Sativa*, brings a new hope to people suffering from drug-resistant epilepsy. However, the hydrophobic character of this compound significantly lowers its clinical efficiency. One of the promising methods of this substance bioactivity increase is delivery through the skin tissue. In this article, a new type of advanced transdermal systems based on chitosan and ZnO nanoparticles (NPs) has been developed according to Sustained Development principles. The chemical modification of the biopolymer confirmed by FT-IR method resulted in the preparation of the material with great swelling abilities and appropriate water vapor permeability. Obtained nanoparticles were investigated over their crystalline structure and morphology and their positive impact on drug loading capacity and cannabidiol controlled release was proved. The novel biomaterials were confirmed to have conductive properties and not be cytotoxic to L929 mouse fibroblasts.

## 1. Introduction

Epilepsy, a neurological disease, affects more than 70 million people all over the world and relates mostly to spontaneous seizures and shivering. It is also associated with various other symptoms, both psychiatric and somatic. Seizures may start in only one part of the brain, but they may also spread to its other parts and result in various body malfunctions, even life-threatening ones. Until now, numerous handling methods have been developed. The most common antiepileptic drugs way of action is to mitigate seizure. Nevertheless, around 30% of patients still suffer from the treatment-resistant epilepsy, which seriously affects the quality of their lives [1,2,3]. Epileptic seizures may be caused by numerous factors including brain-related ones such as tumors and stroke, as well as alcohol, hypoglycemia, and others. Noteworthy, this illness origin for many people still remains unknown. In recent years, it has been proved that long-term administration of antiepileptic drugs does not resolve the problem. Thus, both scientists and medics are focused on alternative substances which may help to overcome the limitations of currently used therapy methods [1,2,3]. One of the most promising chemicals is cannabidiol (CBD)—a natural compound obtained from *Cannabis sativa*. Importantly, on the contrary to tetrahydrocannabinol, it does not cause any euphoric effects, thus it may be administrated even in high doses [4]. Until now, there have been numerous clinical studies proving its efficiency without showing any disturbing side-effects, and it is well-tolerated by patients. Importantly, its healing effect has been proven in podiatry. Noteworthy, its mechanism of action is not fully understood yet, but it is known that this terpenophenol interacts with some non-endocannabinoid systems and its biological activity depends on its concentration. Human studies confirmed that CBD is well-tolerated by patients at both low and high doses, even up to 1500 mg/day, and its administration, both acute and chronic, does not cause any significant changes in central nervous system functionality. However, it has been shown that this compound may have immunosuppressive properties due to its reductive effect on Interleukin-8 and -10 biosynthesis as well as the possibility of lymphocyte apoptosis induction [5,6,7,8,9].

Cannabidiol has neuroprotective, antioxidant, and anti-cancer properties. Until now, this compound was used to treat various skin diseases such as atopic dermatitis, psoriasis, and acne, as well as chronic pains, depression, insomnia, anxiety rheumatoid arthritis, etc. CBD has also been shown to exhibit antimicrobial effect against Gram-positive bacteria such as *Staphylococcus aureus*. Therefore, cannabidiol is not only gaining popularity in medicine and pharmacy but also it has a great potential as a cosmetic formulation ingredient [5,9,10,11,12,13,14,15].

Cannabidiol is very poorly soluble in water, which significantly reduces its bioavailability that is only 13–17% when administrated orally. On the other hand, administration via aerosolization is characterized by bioavailability at the level above 30%. Therefore, it is very hard to obtain a desired concentration of this substance in plasma and consequently clinical efficacy. Until now, only few different drug formulations were developed. Currently, there are two main CBD-based medicines available on the market, namely Epidiolex^®^ and Volcano^®^. However, due to the unsatisfactory pharmacokinetics of cannabidiol administration via gastroenteric route, alternatives must be developed. Although one of the common approaches is to obtain a new derivative which would be characterized by higher bioavailability due to the increased hydrophilicity, one must acknowledge that any change in the chemical structure of the compound may cause deterioration of its biological properties [10,11,12,13,14,15,16].

Transdermal delivery is a very promising alternative to traditional drug administration routes and has many advantages over them, especially due to the fact that the medicine does not have to pass gastrointestinal system and misses so-called first pass liver effect. Moreover, it enables significant reduction in the active substance dosage, eliminates discomfort associated with pill swelling or receiving injections and allows its immediate administration termination if needed. It also prevents from the drug degradation due to the pH of gastric fluids. Nevertheless, the efficiency of use of transdermal systems depends on the active substance molecules penetration across the skin barrier (stratum corneum) and possibility of the therapeutic dose transport to the vascular system. Another problematic issue to be overcame is the protective behavior of the skin components as well as the risk of hydrophobic substance accumulation in the tissue. Noteworthy, it enables lipophilic drugs delivery, the bioavailability of which via oral route is greatly limited. Until now, numerus methods for stratum corneum enhanced penetration have been developed such as microneedles, ionophoresis, electroporation, or sonophoresis [16,17].

Currently, scientists are working on functional transdermal delivery systems [17]. The most popular ones are based on polymeric membranes, although the use of such patches may cause skin irritation, which is a highly undesired effect. Therefore, one of the most promising candidates for this type biomedical device is chitosan (CS)—a biopolymer characterized by excellent biocompatibility with skin cells. It can be obtained from various natural sources such as wastes from food industry, insect exoskeletons, or mushrooms and fungi. This polysaccharide exhibits some unique features which can be assigned to its polycationic nature and include lack of cytotoxicity, biodegradability, mucoadhesiveness, and antioxidant, hemostatic or antimicrobial activity. Importantly, chitosan has been shown to interact with cell membrane components, thus increasing its permeability. Chitosan is widely applied in medicine and pharmacy as wound dressings, drug delivery systems, scaffolds, or hemostatic agents. It is also used in wastewater treatment or catalysis [18,19,20,21,22,23,24,25,26,27,28,29]. CS-based transdermal systems can be in various forms such as nanoparticles, microspheres, or membranes. Multiple possibilities of drug immobilization in chitosan matrix result from its capability of both chemical and physical hydrogels formation. Most of them are obtained via crosslinking reaction. The preparation of dense, three-dimensional structure enables efficient active substance incorporation followed by its controlled release in time due to the matrix degradation or diffusion effects. However, a poorly chosen crosslinking agent such as commonly used glutaraldehyde may result in side-effects [30]. Therefore, there is a strong need for more suitable chitosan-based transdermal systems development. A very important feature is the maintenance of the free amino groups of chitosan deacetylated mers, which are responsible for prolonged interactions between the transdermal patch and patient’s skin surface proteins [30]. Noteworthy, there is an increasing attention considering biomaterials preparation using CS of fungal origin due to its superior physiochemical and biological properties combined with more sustained production process [31,32]. Recently, a lot of attention is focused on the hydrogel systems modification with various nanoparticles which significantly affect the drug loading and release behaviors and make them more suitable for stimuli-responsive applications [30,33,34,35,36,37].

In this article, an attempt was made to obtain a novel type of transdermal, crosslinked chitosan-based patches functionalized with ZnO nanoparticles designed for controlled CBD delivery. Chitosan is a well-known substrate used for biomaterials application. Generally, this biopolymer is obtained from crustacean shells during deacetylation process under harsh conditions and consists of three main steps, namely demineralization by inorganic acid, deproteinization, and alkali deacetylation. In the next step, CS is extracted and precipitated. However, the ready product physicochemical properties are often ambiguous and may be contaminated by allergic proteins coming from shrimp, crab, or squid exoskeletons. A strictly defined chemical composition of the raw materials for biomedical devices is crucial for their future application. Thus, for the preparation of new transdermal delivery systems, biopolymer obtained from *Aspergillus niger* was used. The ready biomaterials were investigated over their chemical structure, morphology, and swelling properties. The CBD release into simulated body fluids was investigated. Finally, the XTT assay carried out on L929 confirmed their lack of cytotoxicity showing their great potential in this use.

## 2. Materials and Methods

### 2.1. Materials

Chitosan produced from *Aspergillus niger* (Deacetylation degree (DD) = 92%, determined by FT-IR method; Mw = 890,000 g/mol; and 100–300 cps), fluorescein, L-aspartic, and L-glutamic acid were purchased from PolAura, Dywity, Poland. Propylene glycol, isopropanol, methanol (HPLC grade), (CH_3_COO)_2_Zn∙2H_2_O, CaCl_2_·2H_2_O, MgSO_4_, KCl, KOH, ethanol (96%, 99.9%), KHPO_4_, Na_2_HPO_4_, NaHCO_3_, NaCl, HCl, NaOH, mouse L929 (commercial cell line from mouse C3H/An connective tissue for research purpose only, The European Collection of Authenticated Cell Cultures (ECACC)), Dulbecco’s Modified Eagle Medium (DMEM) with glucose content cell culture medium, antibiotics (streptomycin/penicillin), fetal bovine serum (FBS), phosphate buffer solution (PBS), trypsin with EDTA, and Triton-X-100 were purchased from Sigma Aldrich, Poznań, Poland. For the study, pure cannabidiol of HPLC confirmed purity was used, purchased from Dobre Konopie, Mysłowice, Poland.

### 2.2. Methods

#### 2.2.1. Sample Preparation

Zinc oxide nanoparticles were prepared by direct precipitation method using zinc acetate dihydrate and potassium hydroxide as precipitating agent. Briefly, two aquatic solutions were prepared (2.208 g of zinc acetate per 100 cm^3^ of distilled water and 6.27 g of potassium hydroxide per 100 cm^3^ of distilled water). Alkali solution (20 cm^3^) was added dropwise using peristaltic pump (flow rate = 0.5 cm^3^ per min) into the zinc acetate with constant mixing on magnetic stirrer at 80 °C until reaching pH = 11 and white precipitation forming (Zn(OH)_2_). The pH value was determined by Elmetron electrode, Elmetron, Zabrze, Poland. In the next step, zinc hydroxide was filtered on Buchner funnel, and rinsed with distilled water and absolute ethanol followed by air-drying. Finally, the semi-product was calcinated using ceramic crucible at 400 °C for 2 h under 1000 hPa in air with a free gases flow. To prepare transdermal systems fungal chitosan (0.5 g) was dissolved in the 30 cm^3^ of water/propanodiol (1:1) solution containing L-aspartic and L-glutamic acid (0.5 g; 0.5 g) with various amounts of ZnO NPs (Table 1) and placed in the microwave oven (power = 800 W) for 3 min. Ready samples were washed out from uncreated residues (until pH = 6.5) and lyophilized. Cannabidiol loading was performed by sorption of different amounts of 0.5 mg of CBD per 1 cm^3^ of 96% ethanol solution.

#### 2.2.2. Physicochemical Properties Study

Chemical structure of the samples was determined by Fourier Transform Infrared Spectroscopy FT-IR using FT-IR Nexus 470 Thermo Nicolet spectrometer, Thermo Fisher Scientific, Waltham, MA, USA. The spectra were collected for lyophilized samples using ATR (attenuated total reflection) adapter. Zinc oxide nanoparticles (50 mg per analysis) were investigated over their crystalline structure by X-ray Diffraction (XRD) method using BRUKER Advanced D8, BRUKER, Zastávka, Czech Republic. UV-Vis spectra were collected with UV-Vis spectrophotometer Agilent 8453, Santa Clara, USA. Samples conductivity was measured by their placing between two platinum plates (1 cm distance, 1 cm^2^ area) using SBF as a reference. Into the measuring cell, simulated body fluid solution was poured at 25 °C. Then, SBF conductivity was measured (control). In the next step, each sample was immersed in the solution and placed between two electrodes. Conductivity was measured by Elmetron conductivity meter. ZnO NPs morphology was investigated by Transmission Electron Microscope (TEM), Jeol, Peabody, MA, USA. For the analysis, nanoparticles were dissolved in methanol, placed on copper mesh with formvar-modified surface and left for total evaporation. The methanol was used as a solvent since it evaporates faster than water and enables homogeneous distribution of evaluated NPs during imaging. Image was taken under HT = 80 kV, exposure time 800 ms. The electron dose was 2771.9 e/nm^2^. The samples and composites were evaluated also by Scanning Electron Microscopy, FEI Quanta 650 FEG SEM microscope, ThermoFisher Scientific, Oregon, USA. The curvature and roughness of the sample were determined using Fiji software and Kappa plugin (Open-source software). The elemental composition was investigated by an EDAX^®^ adapter ((X-ray fluorescence method). Morphology of the swollen samples was evaluated using Inverted Optical microscope equipped with epifluorescence adapter, Delta Optical, Zielona Góra, Poland. For this purpose, the samples were swollen with fluorescein methanol solution.

Swelling capacity was investigated by determining the amount of the swelling medium absorbed after 1 h (distilled water and SBF) based on the weight change of the sample. The SD (swelling degree) was calculated using Equation (1):

SD = (W_t_/W_0_) (1)

where SD is the swelling degree, g/g; W_t_ is the sample weight after 1 h, g; and W_0_ is the initial weight of the sample, g.

Porosity of the samples was calculated using Equation (2). For the study, the samples were immersed in 10 cm^3^ of the isopropanol and the volume change was determined.

p = (V_1_ − V_3_)/(V_2_ − V_3_) ∙ 100% (2)

where p is the porosity, %; V_1_ is the initial volume of isopropanol, cm^3^; V_2_ is the volume of isopropanol with immersed sample, cm^3^; and V_3_ is the volume of isopropanol after sample removal, cm^3^.

Water vapor permeability was determined by filling multi plates (1 cm^2^ area) with distilled water (5 cm^3^) and sealing them with each sample. The WVTR was measured based on the amount of evaporated water (weight loss) using Equation (3):

WVTR = (W_t_ − W_0_)/(tA)(g∙m^−2^∙d^−1^) (3)

where W_t_ is the weight after time t; W_0_ is the initial weight; t is the measuring time; and A is the area of the opening of polystyrene well.

To determine mechanical properties of the samples, tensile strength (TS) was measured. For this purpose, the samples were prepared in the form of a “dog bone” by placing swollen hydrogels in the molds printed on 3D Ender printer, Botland, Batlin, Poland, with the dimensions presented in the PN-EN ISO 527: 1998 standard for plastics mechanical properties evaluation followed by lyophilization. The samples of the specified shape (thickness 4.0 mm, measuring part width 10 mm, and overall length 150 mm). The tests were carried out for the samples swollen with simulated body fluid.

#### 2.2.3. Drug Release Study

The in vitro drug release study was carried out under modified sink conditions using simulated body fluid as acceptor medium (pH = 5.5) by the method described by other researchers [36]. The temperature was 37 °C. The experiments lasted six days (until complete drug release for at least one sample). The amount of CBD being released was measured by UV-Vis spectroscopy. Kinetics of the drug release process was using Equations (4)–(7):

(4) $$-\frac{dc}{dt}=kc$$

(5) $$\frac{dc}{dt}=-kc$$

(6) $$\int_{0}^{c_{t}}\frac{dc}{c}=-\int_{t=0}^{t}k\;dt$$

(7) $$\ln\;\frac{c}{c_{0}}=-kt$$

where *c* is the concentration, mg/100 mL; *t* is the time, min; and *k* is the constant, 1/min.

#### 2.2.4. Cytotoxicity Study

Quantitative cytotoxicity of the prepared samples was performed by XTT assay method, which is a standard procedure for biomedical devices evaluation. For this purpose, L929 mouse fibroblasts were cultured under standard conditions (37 °C, 5% CO_2_, 98% humidity) using DMEM cell culture medium supplemented with 10% FBS and 1% antibiotic. The percent of viable cells was calculated according to the producer protocol. As a negative control, 1% Triton-X-100 solution (cell lysing agent) was used. The qualitative cytotoxicity assessment was performed by morphology study under inverted microscope by direct method. For this purpose, 10 mg of each sample were placed in each hole of the 24-well plates. All experiments were carried out using sterile plastics (vials, 24-well plates, 96-well plates, centrifuge tubes, etc.) purchased from GenoPlast, Rokocin, Poland. All of the experiments were carried out under sterile conditions (under laminar chamber). The components in the form of the solution were sterilized using 0.22 µm syringe PTFE filters, Chemland, Stargard Szczeciński, Poland. The samples were sterilized using 70% ethanol and UV radiation. The equipment was sterilized using autoclave and/or 70% ethanol.

Statistical analysis was determined using Microsoft Excel software. A *p* < 0.05 value was considered as statistically significant.

## 3. Results and Discussion

### 3.1. Physicochemical Properties Study

**Figure 1:**
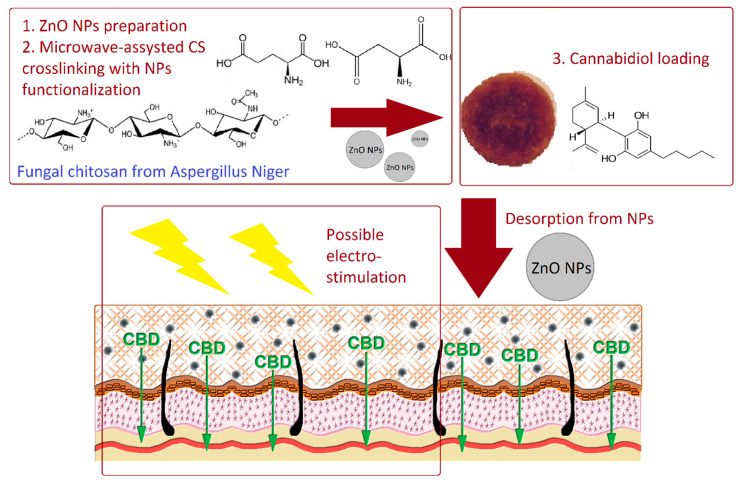
General scheme for transdermal delivery system preparation and potential application.

Figure 1 presents the general strategy for novel biomaterials development and their potential application in cannabidiol transport through skin tissue (Figure 1).

**Figure 2:**
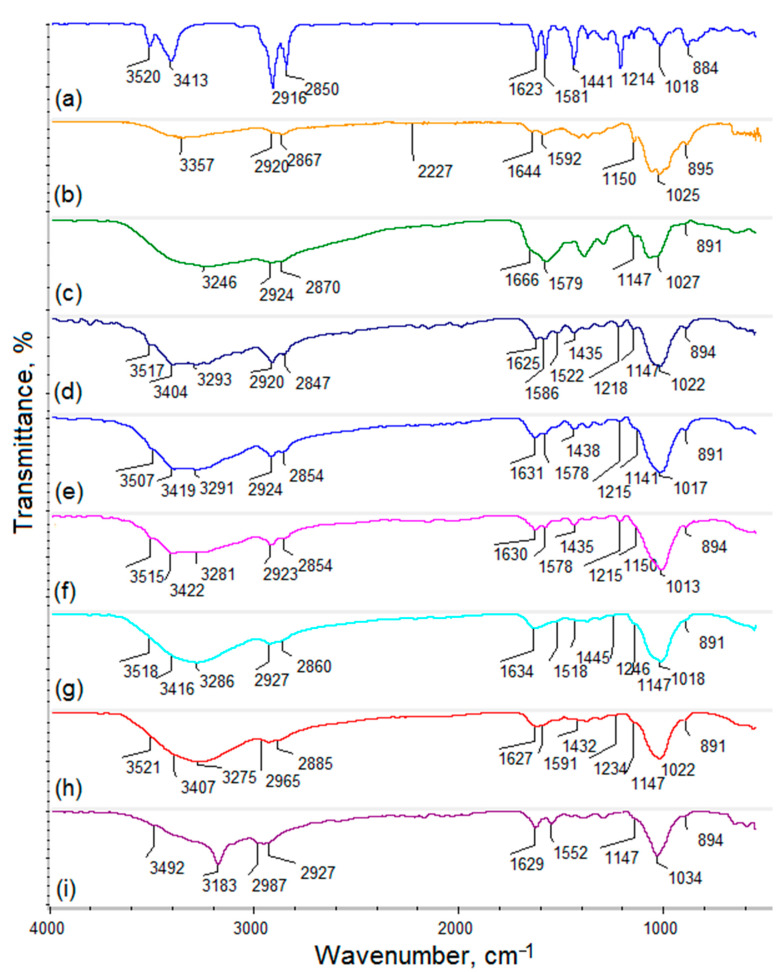
FT-IR spectra:(**a**) cannabidiol; (**b**) pure chitosan; (**c**) chitosan crosslinked with L-aspartic and L-glutamic acid; (**d**) CS-ZnO-1; (**e**) CS-ZnO-2; (**f**) CS-ZnO-3; (**g**) CS-ZnO-4; (**h**) CS-ZnO-5; and (**i**) CS-ZnO-6.

Figure 2 shows FT-IR spectra of the fungus chitosan, CBD, and ready transdermal delivery systems loaded with active substance. To investigate loading capacity, the samples were loaded with different amounts of cannabidiol dissolved in pure ethanol. Pure cannabidiol exhibits some typical bands at 3520 cm^−1^, coming from OH groups, and 3413 cm^−1^. Bands with the maximum at 2916 and 2850 cm^−1^ come from aliphatic groups (-CH_3_ and -CH_2_-). Bands with the maximum at 1623 and 1581 cm^−1^ come from stretching vibrations of the C=C groups and -CH_2_- bending at 1441 cm^−1^ [38]. Raw chitosan spectrum shows two overlapping bands (O-H and N-H stretching) at 3357 cm^−1^. The bands coming from -CH_3_ and -CH_2_- groups were collected at 2920 and 2867 cm^−1^. The band with the maximum at 1644 cm^−1^ comes from amide I bonds. The presence of free amino groups present in deacetylated glucosamine mers are visible at 1592 and 1150 cm^−1^. Finally, the bands coming from glycosidic bonds linking glucosamine and N-acetyglucosamine mers inside the polymer are visible at 1050 cm^−1^, whereas the band typical for glucopyranose ring is present at 895 cm^−1^. The spectrum of the fungal chitosan crosslinked with L-aspartic and L-glutamic acid show some significant changes when compared to the raw chitosan mainly due to the increase in amide bonds intensity, which proves covalent bonds formation between –COOH functional groups of the amino acids and free NH_2_ of the biopolymer (1666, 1629, 1627, 1634, 1630, 1631, and 1625 cm^−1^). There is no band coming from ester bonds and the band coming from free hydroxyl groups is still present. The crosslinked material structure also contains free carboxylic groups coming from not fully incorporated amino acids, which proves the band with the maximum at 3246 cm^−1^. For the samples modified with different amounts of ZnO NPs, some additional bands are visible coming from –OH and their intensity is correlated with the quantity of the nanoadditive at 3404 (CS-ZnO-1), 3419 (CS-ZnO-2), 3422 (CS-ZnO-3), 3416 (CS-ZnO-4), 3422 (CS-ZnO-5), and 3492 cm^−1^ (CS-ZnO-6), respectively. The FT-IR analysis confirmed also immobilization of CBD inside the polymeric matrix which proves the presence of bands at 3517, 1435, and 1218 cm^−1^ (CS-ZnO-1); 3507, 1438, and 1215 cm^−1^ (CS-ZnO-2); 3515, 1435, and 1215 cm^−1^ (CS-ZnO-3); 3518, 1445, and 1246 cm^−1^ (CS-ZnO-4); 3521, 1432, and 1234 cm^−1^ (CS-ZnO-5); and 3492 cm^−1^ (CS-ZnO-6). Importantly, for all of the prepared samples, bands proving free NH_2_ groups are visible, which means the preservation of intrinsic biological properties of the pristine chitosan such as adhesiveness resulted in the prolonged skin-patch contact, biocompatibility and ability to increase the cell membrane permeability [30]. The mechanism of the hydrogel production is based on the principle that chitosan has functional groups in its structure capable of covalent bonds formation. As a result of crosslinking reaction, glutamic and aspartic acids, which contain both NH_2_ and COOH groups, form amide bonds between their carboxyl groups and free amino groups of the chitosan. Thus, the hydrogel has more dense structure and does not dissolve in water or any aquatic media as physical hydrogels. The ZnO NPs were incorporated into the polymeric matrix during crosslinking process. The NPs immobilization was possible due to the hydrogen bonds formation and electrostatic interactions.

**Figure 3:**
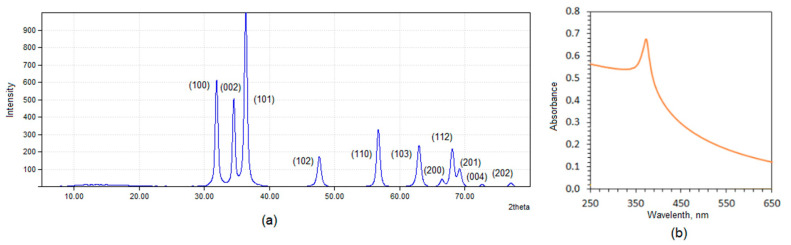
**(a)** XRD analysis of the prepared ZnO NPs; and (**b**) UV-Vis spectrum of the prepared ZnO NPs.

To confirm ZnO nanoparticles preparation, XRD and UV-Vis analyses were carried out. The results given in Figure 3a clearly proves ZnO NPs obtainment since the reflections are in line with other researcher’s data and are typical for this nanomaterial. ZnO nanoparticles have a wurtzite of nanocrystal structure with centro-simmetric character. The structure is composed of Zn cations and O anions. The constants for a crystalline unit are 3.21 and 5.21 Å. UV-Vis spectrum (Figure 3b) exhibits a broad band with the maximum at 380 nm typical for pure ZnO nanoparticles [33,34,35].

**Figure 4:**
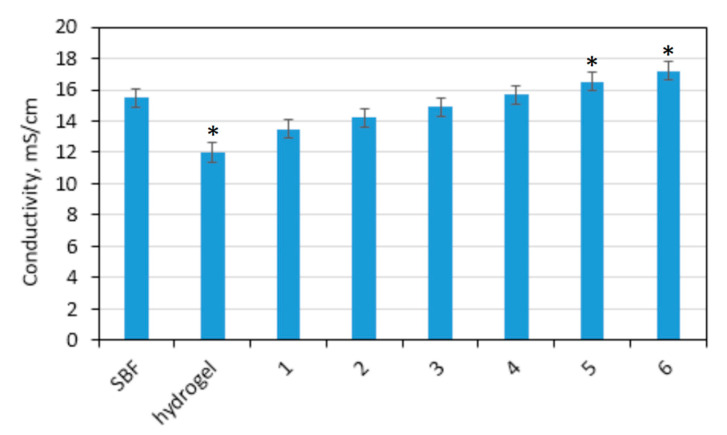
Conductivity of the prepared transdermal systems: (1) CS-ZnO-1; (2) CS-ZnO-2; (3) CS-ZnO-3; (4) CS-ZnO-4; (5) CS-ZnO-5; and (6) CS-ZnO-6. (* *p* < 0.05).

Zinc oxide nanoparticles are known to be semiconductors and can conduct current. Therefore, their addition to the polymeric matrix may result in the enhancement of materials conductivity, which is a very favorable feature in terms of the possibility to perform electro stimulated transdermal delivery. Figure 4 shows conductivity study performed on swollen hydrogel as well as ZnO-doped composites containing different amounts of these NPs and SBF as a reference. It may be noticed that chitosan-based hydrogel has significantly lower conductivity comparing to the simulated body fluid and ions movement inside of the 3D matrix can be hampered. In the case of the nanocomposites, there is a significant difference in the ability to current flow to conduct electricity and it increases with ZnO NPs content. Thus, it may be assumed that the electric field application may result in the increase of skin tissue permeability and CBD molecules would be transported due to the electrophoresis and electroosmosis, which is called an iontophoresis process [30,39].

### 3.2. Morphology Study

**Figure 5:**
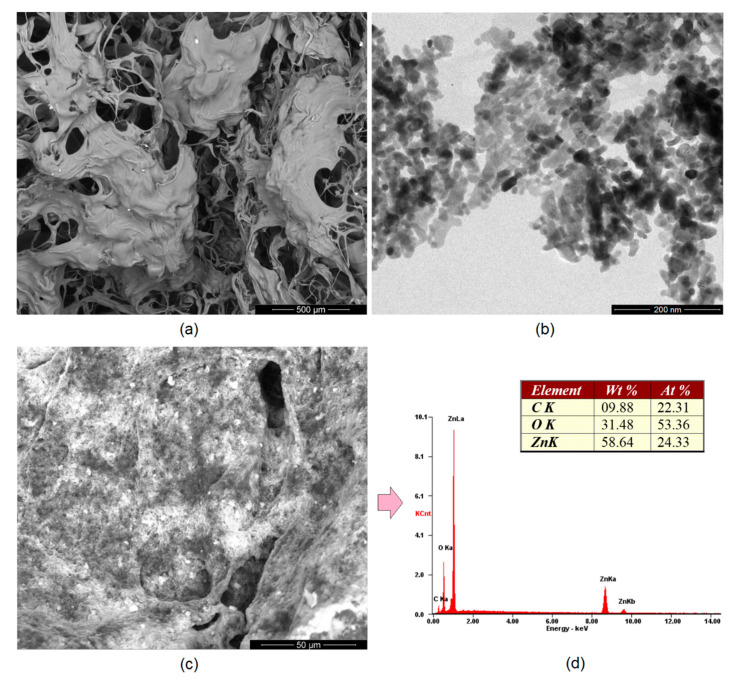
(**a**) SEM microphotograph of the chitosan hydrogel crosslinked with amino acids; (**b**) TEM microphotograph of the prepared ZnO nanoparticles; (**c**) SEM microphotograph of the hydrogel modified with ZnO nanoparticles (CS-ZnO-6); and (**d**) elemental analysis of the sample surface (CS-ZnO-6).

**Figure 6:**
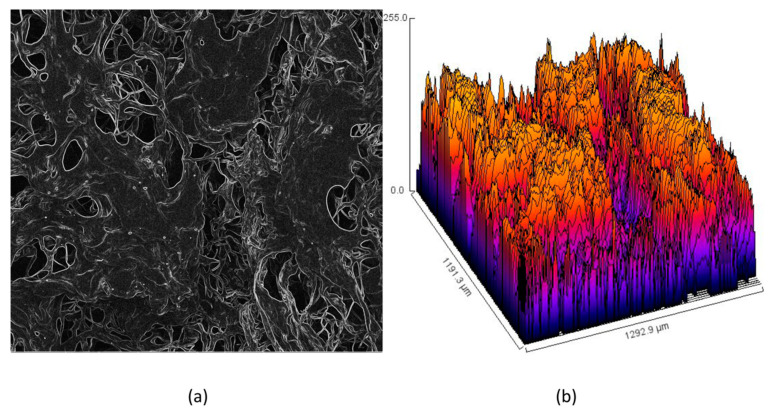
(**a**) Curvature of the chitosan hydrogel; and (**b**) roughness of the chitosan hydrogel.

Appropriate morphology of the material used as a potential transdermal patch is crucial for several reasons. The structure should be highly porous [25,26,27,37]. Importantly, there are many bioinert and biocompatible polymeric devices which could be potentially applied for drug delivery, but because of their very low porosity or even solid character cannot be used for this purpose. Figure 5a shows SEM microphotograph of the unmodified hydrogel. It can be noticed that it has open-pores structure and very high surface area. The pores created interconnected channels which will enable water permeability as well as possibility of biomolecules migration [30]. Such extensive 3D network gives possibility of high amount of various solutions sorption. The pores edges are mild and of petal-like shape and their average size is 100–150 µm. Figure 5b shows TEM microphotograph of the prepared ZnO particles. It may be observed that all of them are in the nano size and below 100 nm. Their shape is round or slightly cubical with 20 nm average diameter. No sharp edges are visible which could potentially irritate skin after placing. No additional substances or residues are visible in the picture. Noteworthy, the results correspond to the data given in Figure 3 and previously reported data [37]. Figure 5c shows SEM microphotograph of the sample modified with ZnO NPs. It can be observed that the NPs are homogeneously distributed inside polymeric matrix and the surface of the material is uniform and not degraded. Figure 5d shows XRF spectrum of the sample surface showing its elemental composition. There are three main elements present, namely carbon, oxygen, and zinc, which proves the purity of the prepared sample as well as the presence of the ZnO NPs in the material. Figure 6 provides details of the lyophilized hydrogel structure (curvature (Figure 6a) and roughness (Figure 6b)). The lyophilized samples before swelling are characterized by a high curvature (average 0.33 μm^−1^). Moreover, their surface is well-developed and of three-dimensional, spacious architecture. Such roughness should provide good adhesion to patients’ skin and is also responsible for good swelling abilities and drug loading capacity.

**Figure 7:**
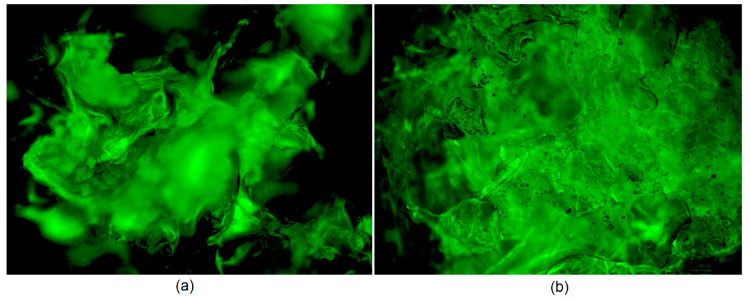
Microphotographs of the samples: (**a**) swollen hydrogel (40×); and (**b**) swollen hydrogel modified with ZnO nanoparticles and immobilized CBD (40×).

To investigate the morphology of the swollen samples, optical microscope with epifluorescence adapter was used (Figure 7). It can be noticed that, even after solution sorption, the samples (empty hydrogel (Figure 7a) and hydrogel modified with ZnO NPs containing CBD (Figure 7b)) are porous and with well-developed surface area. In addition, there is a small difference in the morphology of the sample with and without the addition of NPs. It can be noticed that ZnO-modified polymeric matrix is more curved and rigid, and some NPs covered with CBD are visible, which are quite homogenously distributed.

### 3.3. Swelling Abilities Study

**Figure 8:**
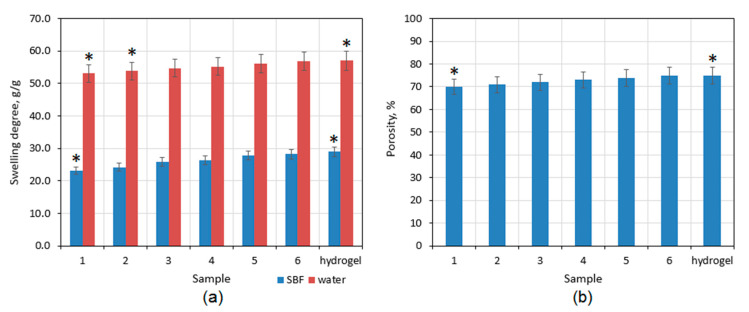
(**a**) Swelling degree of the developed samples: (1) CS-ZnO-6; (2) CS-ZnO-5; (3) CS-ZnO-4; (4) CS-ZnO-3; (5) CS-ZnO-2, and (6) CS-ZnO-1. (**b**) Porosity of the developed samples: (1) CS-ZnO-6; (2) CS-ZnO-5; (3) CS-ZnO-4; (4) CS-ZnO-3; (5) CS-ZnO-2, and (6) CS-ZnO-1. (* *p* < 0.05).

Good swelling properties are typical for hydrogels, which can be defined as three-dimensional, branched structures (Figure 8a). Prepared patches have a highly porous morphology (Figure 5, Figure 6 and Figure 7) and hydrophilic groups in their structure thanks to their modification with amino acids (Figure 2). In general, it is known that hydrogels are superior to other polymeric materials in diffusion-controlled drug delivery systems [17,25,26,27,30,37,39,40]. They have a great affinity to aquatic solution. Noteworthy, swelling degree is different depending on the swelling medium. The average SD for distilled water is 55 g/g, whereas SD for simulated body fluid is approximately twice lower. The highest SD is for unmodified hydrogel, whereas the lowest is for the sample with the highest content of ZnO NPs. Figure 8b shows the porosity of the materials which is at least 70% for all samples. The parameter increases with the decrease of the zinc oxide nanoparticles amount. Nevertheless, the difference between them is quite low. Prepared hydrogels have a dense structure, and, due to chemical crosslinking, they do not dissolve in aquatic medium but are three-dimensional, sponge-like materials which absorb high amounts of water. The term hydrogel in this case refers to the 3D, highly porous structure which can be swollen with aquatic medium.

### 3.4. Mechanical Properties and Water Permeation Study

**Figure 9:**
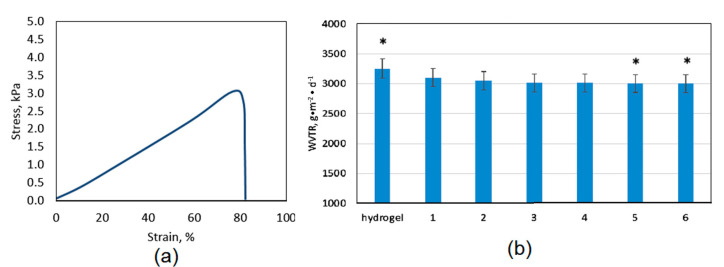
(**a**) tensile strength of the swollen hydrogel; and (**b**) water vapor permeability. (* *p* < 0.05).

**Figure 10:**
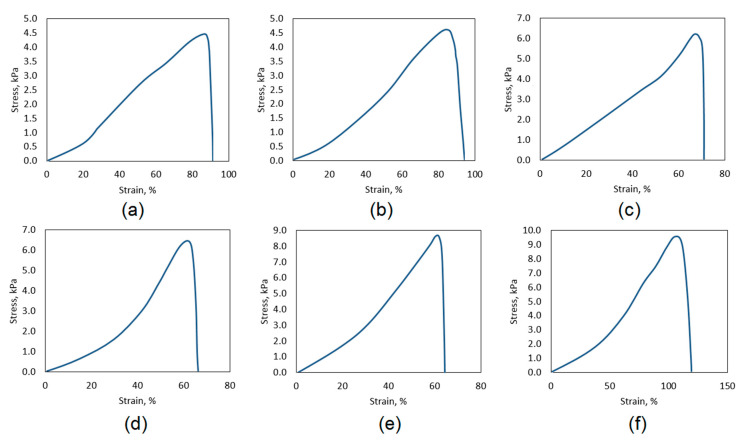
Tensile strength of the hydrogels containing: ZnO (**a**) CS-ZnO-1; (**b**) CS-ZnO-2; (**c**) CS-ZnO-3; (**d**) CS-ZnO-4; (**e**) CS-ZnO-5; and (**f**) CS-ZnO-6.

The systems for controlled drug delivery in the form of a patch should exhibit appropriate mechanical durability to provide sufficient cannabidiol transport through skin tissue without breaking due to the movement or some external factors which could result with the change of bioactive substance release profile [41,42]. The tensile strength of the pure, swollen with SBF hydrogel is almost 3.5 kPa. The mechanical durability is affected among others by the materials porosity. Another parameter which is associated with hydrogels morphology and chemical structure is water vapor transmission rate (Figure 8b). Noteworthy, the polymeric patch which is used as transdermal CBD delivery system should not limit natural process of water molecules migration. WVTR parameter for healthy skin oscillates around 3000 g∙m^−2^∙day^−1^. Therefore, the transdermal patch should be characterized by water permeation of at least 2500 g∙m^−2^∙day^−1^. Figure 8b shows that all of the samples exhibit excellent permeability for H_2_O molecules which is caused by their hydrophilic character and open-pores, spacious architecture [30,41]. Chitosan hydrogels depending on the manufacturing method may have various durability; nevertheless, in most cases, their mechanical properties are mediocre or even not satisfactory [42,43]. Thus, one of the most effective methods is doping with nanoparticles or mixing with other polymers and inorganic components. One may observe that, comparing TS of the unmodified hydrogel (Figure 9a) to the samples containing ZnO nanoparticles (Figure 10), a significant increase in their durability is visible. Figure 10 shows that there is a correlation with the amount of ZnO NPs and TS. The best mechanical properties were obtained for the samples CS-ZnO-5 and CS-ZnO-6. One may notice that zinc oxide NPs addition resulted in more than twofold increase in tensile strength. The TS of the nanocomposites should provide appropriate conditions for their applications as polymeric patches for transdermal CBD delivery [30].

### 3.5. Cannabidiol Controlled Release Study

**Figure 11:**
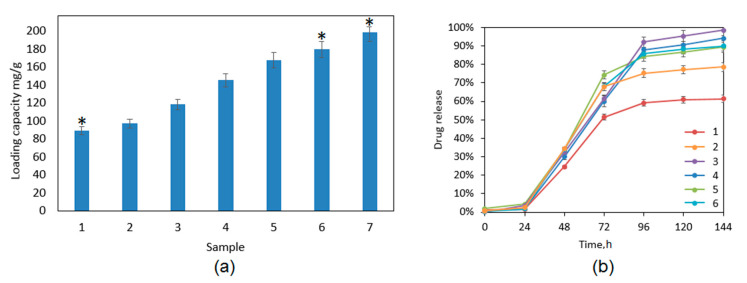
(**a**) Drug loading capacity: (1) hydrogel; (2) CS-ZnO-1; (3) CS-ZnO-2; (4) CS-ZnO-3; (5) CS-ZnO-4; (6) CS-ZnO-5; and (7) CS-ZnO-6. (**b**) Cannabidiol release profile in time (six days): (1) hydrogel; (2) CS-ZnO-1; (3) CS-ZnO-2; (4) CS-ZnO-3; (5) CS-ZnO-4; and (6) CS-ZnO-5. (* *p* < 0.05).

**Figure 12:**
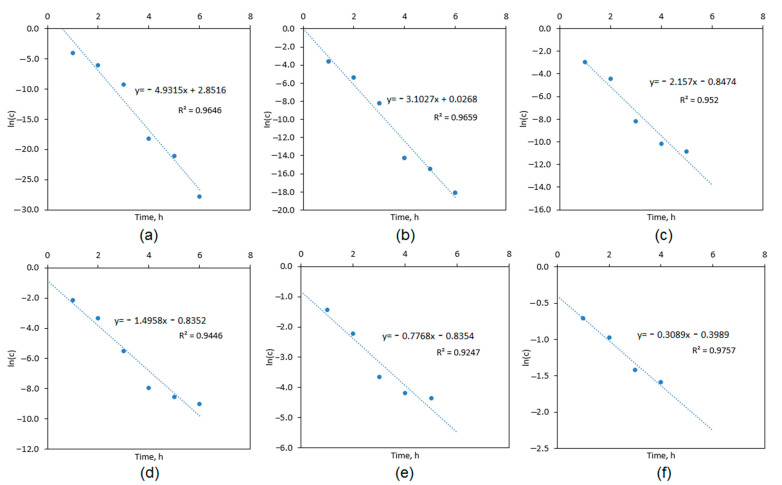
Cannabidiol release kinetics study: (**a**) hydrogel; (**b**) CS-ZnO-1; (**c**) CS-ZnO-2; (**d**) CS-ZnO-3; (**e**) CS-ZnO-4; and (**f**) CS-ZnO-5.

Figure 11a presents drug loading capacity for the developed samples. The CBD loading efficiency is correlated with the chemical composition of the biomaterials and depends on the ZnO NPs content. There are a few alternative drug release mechanisms, which are a consequence of the physicochemical properties of the polymeric matrix used for transdermal systems preparation. When a non-degradable carrier is applied, e.g., PDMS (polydimethylsiloxanes), the active substance molecules leave it due to the diffusion process [37]. When a degradable material is used, the drug release may occur due to the erosion of the matrix or hydrolysis of the chemical bonds. Until now, it has been proven that the use of additional components may help to control the release process due to the increase of drug solubility or changing the materials morphology [17,30,37]. The use of nanoparticles is known to have good sorption properties due to their high specific surface. Figure 11b shows drug release profile. The studies were carried out under sink conditions using simulated body fluid as an accepting medium. It may be noticed that, during first 24 h, no burst release effect is observed, which is a highly desired property. In the case of all samples, linear drug outflow is observed during 72 h. The exception was sample ZnO-6, where CBD was leaving the matrix in an uncontrolled and immeasurable manner due to the high increase in the accepting solution turbidity (due to that, the sample is not presented in Figure 11b and Figure 12). After that period of time, some changes between the patches are visible. Crosslinked hydrogel released less than 60% of the CBD after 144 h (six days) and the release profile after the third day plateaued. On the other hand, nanocomposite transdermal systems released at least 20% more of the active substance comparing to the hydrogel alone. Importantly, for Sample 3, the complete release was achieved, and the process was linear until 96 h, and then the release rate decreased. Prolonged outflow of the CBD was obtained for all samples containing different amounts of ZnO NPs, which can be caused by possible adsorption of the CBD molecules on their surface followed by their desorption [36,37]. There is a correlation between the architecture of the materials and their drug release abilities. The drug release behaviors in this case were mostly determined by the ZnO NPs content, porosity, and swelling abilities. There was no significant effect of polymeric matrix degradation in time leading to the accelerated drug outflow.

The kinetics study is given in Figure 12. The drug release profile occurs according to the first-order kinetics, which is typical for hydrogels. It is known that the active substance leaves a polymeric matrix mostly due to the diffusion process or materials degradation/erosion [17,30,37]. The unmodified hydrogel released the lowest total amount of CBD and at the highest speed rate. The samples containing ZnO exhibited more sustained cannabidiol release in prolonged manner. Such results show that the process depends not only on the porosity of the materials and their ability of substance removal due to the water molecules penetration but also on desorption from the ZnO NPs surface [35,36]. Noteworthy, CBD release due to its hydrophobic nature is generally impeded under human-like conditions (SBF fluid) but CS-based matrix modification with zinc oxide nanoparticles resulted in the significant improvement of pharmaceutically relevant parameters such as drug loading capacity and controlled release and may contribute to the more efficient CBD transdermal administration.

### 3.6. Cytotoxicity Study

**Figure 13:**
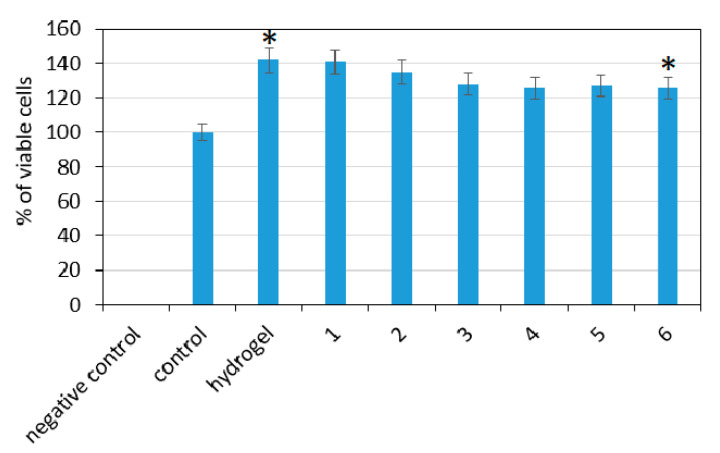
XTT assay carried out on L929 mouse fibroblasts after 48 h: (1) CS-ZnO-1; (2) CS-ZnO-2; (3) CS-ZnO-3; (4) CS-ZnO-4; (5) CS-ZnO-5; and (6) CS-ZnO-6. (* *p* < 0.05).

To verify biological properties of the potential transdermal systems for CBD delivery, XTT tests for quantitative cytotoxicity determination were performed. The assay principle is based on the ability of viable cells to reduce XTT to its orange-colored derivative by cellular enzyme. Figure 13 presents the results for each sample. As a control, fibroblasts cultured in pure cell culture medium were used. According to ISO 10993-5 norm, the sample is considered to by cytotoxic if less than 70% of cultured cells are alive, compared to control. It may be observed that all of the evaluated samples did not cause negative effect to cell viability since, compared to the control (cells cultured without any additive under standard conditions), it was higher in each case. Th highest number of alive cells was in the cases of the pure hydrogel and the sample containing 0.5% of ZnO NPs. A small decrease was noticed with the increasing amount of the ZnO nanoparticles content; nevertheless, this effect can be omitted since the number of living cells was still higher than 100%. Such results show that the samples do not cause any indirect cytotoxicity to L929 mouse fibroblasts cells, which are typically used to study the biological properties of biomedical devices [30].

**Figure 14:**
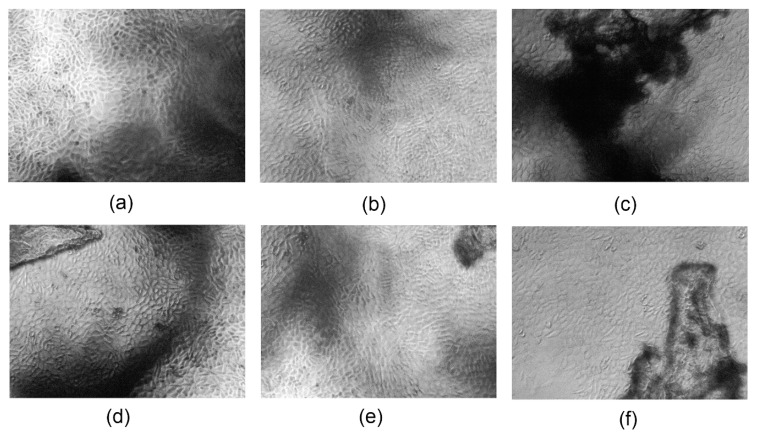
Qualitative cytotoxicity assessment of the developed samples on L929 mouse fibroblasts after six days of cell culture (40× magnification): (**a**) CS-ZnO-1; (**b**) CS-ZnO-2; (**c**) CS-ZnO-3; (**d**) CS-ZnO-4; (**e**) CS-ZnO-5; and (**f**) CS-ZnO-6.

To further investigate the biocompatibility of the samples, direct cytotoxicity test was performed to verify cells morphology, detachment from the cell culture flask bottom, their vacuolization, or some disruption in cell membrane integrity. The results are shown in Figure 14. All of the tested biomaterials exhibited great biosafety since no negative cell–material interactions were noticed after six days of cell culture. After this period of time, mouse fibroblasts, which are the most common cells of the connective tissue, formed a uniform layer and no reduced growth was spotted. There are no or a few intracytoplasmatic granules visible as well as cell under lysis process. In addition, it may be observed that the cells are tightly connected together and there are no free areas between them. The fibroblasts have normal, spindle-like morphology and no abnormalities can be observed. There are no rounded cells. The morphological qualitative assessment showed that the biomaterials have a zero-grade cytotoxicity according to ISO standard for biomedical devices. Taken together, both studies (indirect and direct) confirmed the biosafety of the developed transdermal systems and their potential in the future application in controlled drug delivery through skin tissue [17,30,37,39].

**Figure 15:**
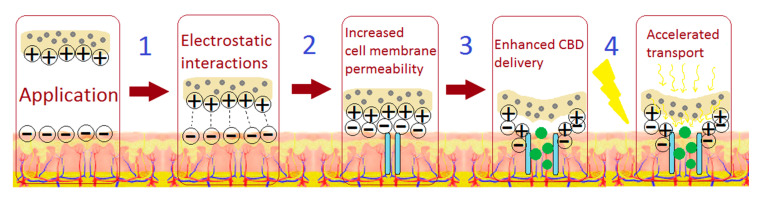
Possible applications of the stimuli-responsive transdermal systems in CBD delivery.

Based on the obtained results, Figure 15 presents possible applications of the newly developed stimuli-responsive transdermal systems for controlled CBD delivery, namely by traditional placing of the patch on skin or with the use of electric field (iontophoresis) [42].

## 4. Conclusions

This article deals with the development and evaluation of novel transdermal delivery systems dedicated to an alternative method of CBD administration. As a starting material, fungi-derived chitosan was used, which was further modified with ZnO NPs of spherical shape, average 20 nm diameter, and Wurtzite crystal structure, as confirmed by XRD and TEM. The materials exhibited superior porosity, as visualized by SEM and fluorescent microscopy, and excellent swelling properties. The addition of zinc oxide nanoparticles resulted in its physicochemical properties enhancement in terms of mechanical durability, drug loading capacity, and prolonged release according to first-order kinetics mechanism. The ZnO presence also had a small negative effect on porosity, which did not result in the materials properties deterioration, such as water vapor permeation. Additionally, the nanocomposites had conductive properties, which gives the possibility of their application in iontophoresis. The cytotoxicity of prepared materials was evaluated by both indirect and direct methods (XTT assay and morphology study), and both confirmed the good biocompatibility of transdermal systems with skin cells (L929 mouse fibroblasts). The presented data show great potential of newly obtained polymeric systems for various methods of CBD delivery to patients suffering from treatment-resistant epilepsy.

## Figures and Tables

**Table 1:** Transdermal delivery systems description.

Sample	CS, g	L-Asp; L-Glu, g	ZnO NPs, wt.%	MW Power, W	Time, min
hydrogel	0.5	0.5;0.5	0.0	800	3
CS-ZnO-1			0.5		
CS-ZnO-2			1.0		
CS-ZnO-3			1.5		
CS-ZnO-4			2.0		
CS-ZnO-5			2.5		
CS-ZnO-6			3.0		

## Data Availability

The data presented in this study are available on request from the corresponding author.
